# Source code and simulation data for the prediction of the electrodeposition mechanism of nanostructured metallic coatings

**DOI:** 10.1016/j.dib.2023.109269

**Published:** 2023-05-26

**Authors:** G. Rosano-Ortega, M. Bedolla-Hernández, F.J. Sánchez-Ruiz, J. Bedolla-Hernández, P.S. Schabes-Retchkiman, C.A. Vega-Lebrún, E. Vargas-Viveros

**Affiliations:** a Popular Autonomous University of the State of Puebla (UPAEP), Puebla, Mexico; b Tecnológico Nacional de México / IT Apizaco, Apizaco, Tlaxcala, Mexico; c Institute of Physics, National Autonomous University of Mexico (UNAM), CDMX, Mexico; d Autonomous University of Baja California. Architecture and Design Faculty, Ensenada, BC

**Keywords:** Programming in Cuda®, Quantum chemistry, Electrochemistry, Nanocoatings, Interdiffusion

## Abstract

This data article presents a simulation model based on quantum mechanics and energy potentials for obtaining simulation data that allows, from the perspective of materials informatics, the prediction of the electrodeposition mechanism for forming nanostructured metallic coatings. The development of the research is divided into two parts *i*) the formulation (Quantum mechanical model and Corrected model for electron prediction; using a modified Schrödinger equation) and *ii*) the implementation of the theoretical prediction model (Discretization of the model). For the simulation process, the finite element method (FEM) was used considering the equation of electric potential and electroneutrality with and without the inclusion of quantum leap. We also provide the code to perform QM simulations in CUDA®, and COMSOL® software, the simulation parameters, and data for two metallic arrangements of chromium nanoparticles (CrNPs) electrodeposited on commercial steel substrate. (CrNPs-AISI 1020 steel and CrNPs-A618 steel). Data collection shows the direct relationship between applied potential (*V_DC_*), current (*A*), concentration (*ppm*), and time (*s*) for the homogeneous formation of the coating during the electrodeposition process, as estimated by the theoretical model developed. Their potential reuse data is done to establish the precision of the theoretical model in predicting the formation and growth of nanostructured surface coatings with metallic nanoparticles to give surface-mechanical properties.

**Specifications Table**

**Table:** 

Subject	*Materials Science*
Specific subject area	*Computational Materials Science, and Surfaces, Coatings, and Films* Numerical-computational analysis of nanostructured surface coatings electrodeposition mechanism for coating-substrate arrangements of metallic materials.
Type of data	Table Picture Graph Figure Code
How the data was acquired	Simulations for data acquisition were performed using a theoretical-computational model applying a modified Schrödinger equation with equations of quantum mechanics (QM), potential energy, and electrochemistry. For the simulation process, the finite element method (FEM) was used considering the equation of electric potential and electroneutrality with and without the inclusion of quantum leap. The models were programmed in CUDA® and COMSOL® programs and executed in supercomputing units of the National Supercomputing Laboratory (LNS- Conacyt) using 10 parallel computing units with Xenon E processors, with NVIDIA Gforce OVX cards with a capacity of 1 TB of operations in one nanosecond.
Data format	Raw data (.txt) Analyzed (.csv)
Description of data collection	The electrodeposition process conditions used in simulations were 0.3 A, 10-15 V_DC_, and 50, 100 ppm of CrNPs to be coated on a flat surface of 1 cm^2^ of A618 and AISI 1020 plates of steel at residence times of 5, 10, and 15 minutes Theoretical data for nanostructured material, used like contributing material, were chromium nanoparticles with the average size of 40 nm, dispersion of ± 5 nm, apparent spherical shape, and oxidation states Cr^0^ and Cr^3+^.
Data source location	*Institution:* Popular Autonomous University of the State of Puebla *City/Town/Region:* Puebla *Country:* Mexico
data accessibility	Repository name: GitHub Direct URL to data: https://github.com/Paco1901/Schrodinger_electrochemistry.git
Related research article	M. Bedolla-Hernández, G. Rosano -Ortega, FJ Sánchez-Ruiz, J. Bedolla-Hernández, PS Schabes-Retchkiman, CA Vega- Lebrún, Electrodeposition mechanism of chromium nanoparticle coatings: Modeling and experimental validation, Chem. Eng. Sci. 252 (2022) 117291. https://doi.org/10.1016/j.ces.2021.117291*.*

## Value of the Data


 

• These data are useful because allow to understand the electrodeposition mechanism in intermetallic systems and visualize the formation and growth of nanostructured coatings where the input material is nanoparticles.
• From these data can benefit professionals and researchers in thin films and coatings areas.
• These data and the source codes generated can be used for further insights and development of experiments because a variation of the parameters in the source codes can also be carried out, allowing the analysis of cases of particular interest that involve this electrodeposition mechanism.
• That allows researchers to establish the necessary electrodeposition parameters (voltage, current, concentration, and residence time) for the experimental deposition of nanostructured metallic coatings, enabling the development of a sustainable electrodeposition process.

## 1 Objective

Provides the predictive part of the electrodeposition mechanism for the fabrication of nanostructured surface coatings with metallic nanoparticles, determines the presence and resulting morphology, and determines the deposition and electrodeposition energies, showing interdiffusion as the form of adhesion of the filler material to the substrate. The data and codes may apply to metallic filler materials and substrate arrangements, opening the experimental application perspective.

## 2 Data Description

**Fig. 1:**
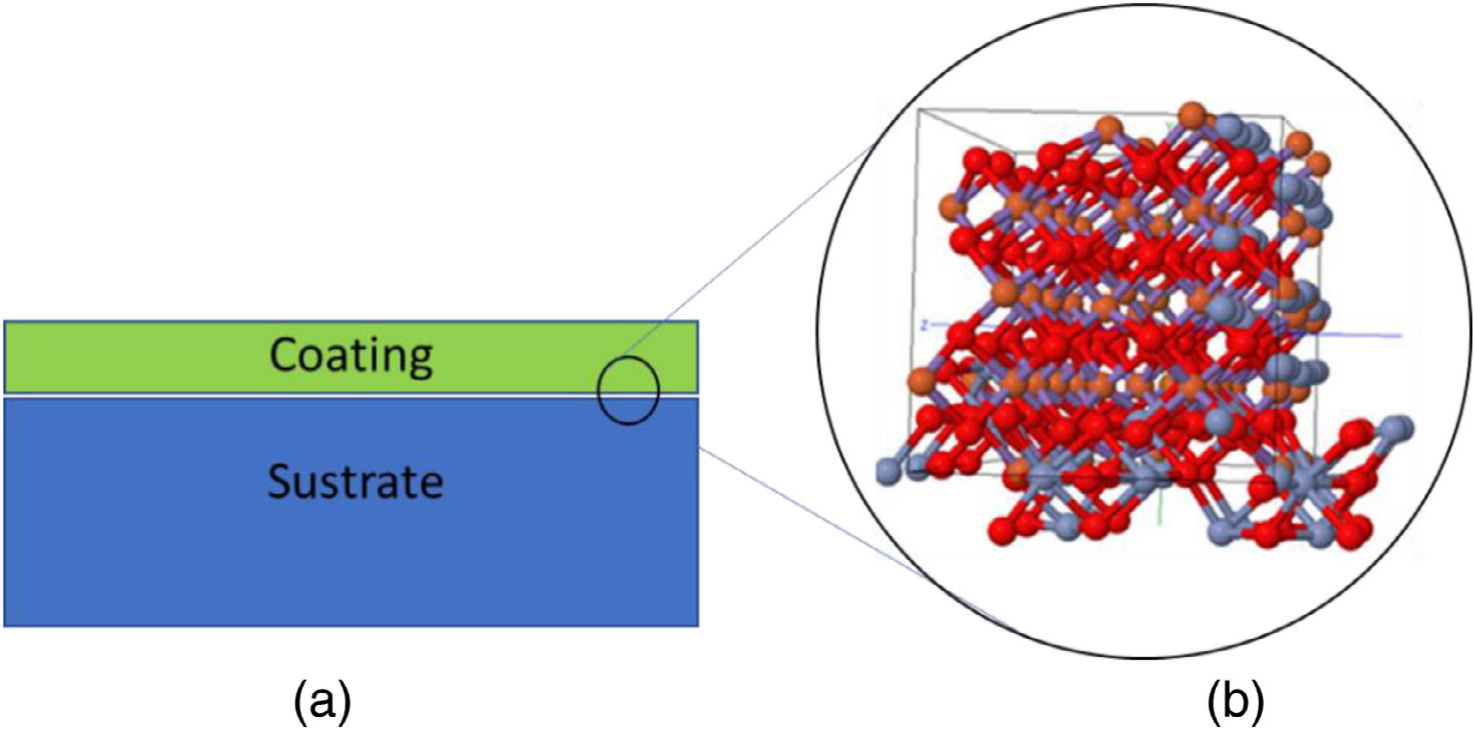
Coating-substrate arrangement.

The data published in this article includes the source codes implemented in the CUDA® and COMSOL® programs to analyze the electrodeposition mechanism of nanostructured surface metallic coatings, from the perspective of materials informatics [1]. The developed model uses electrochemical potentials to implicitly describe the atomic interaction of the surface atoms of a nanoparticle of radius *r* ≈ 40 nm with the respective atoms on the surface of the substrate. In contrast, the electrochemical deposition (absorption) capacity is controlled by electric potential. A subgroup of atoms is identified in a standard zone or interface that shows the presence of interdiffusion during the coating formation process. Fig. 1 shows the arrangement used in the computational simulations.

Fig. 1(a) corresponds to the structure of the array components. The system is intermetallic, constituted in the coating by nanoparticles of chromium Cr^0^ and Cr^3+^ and, as a substrate, a commercial steel plate, according to Table 1. Fig. 1(b) is the *ball and stick model* obtained from the simulations for the resulting structure at the coating-substrate interface, where the presence of interdiffusion is observed, which enhances the adhesion of the coating to the substrate.

In summary, codes source (point 1.1), modify and view files (point 1.2), and the data obtained from simulations (point 1.3) from the Quantum Mechanics (QM) perspective, using a model developed combining the Schrödinger- Lenard -Jones-Nernst-Planck equations and discretized by the finite element method (FEM), are presented in detail below [[3], [4], [5], [6]].

### 2.1 Drivers for Modifying CUDA® Files

There are different free access programming packages, such is the case of those offered by NVIDIA GPU, which were implemented to solve the quantum chemistry equations. The programming language uses the video cards linked to the systems as a means of processing. computer mechanics through the CUDA language, which is a multiplatform language, the drivers for the execution of the programs with *.cu and *.pdb extension can be obtained from the following link for free.

Go to https://developer.nvidia.com/cuda-toolkit and download:

- NVIDIA CUDA-X GPU- Accelerated Libraries for the execution of all the codes located at https://github.com/Paco1901/Schrodinger_electrochemistry.git. The extension identifies all executable files *.cu [6]:
  ○ ***Schrondiger_deposition.cu*** (Equation to predict the deposition of nanostructured material)
  ○ ***Schrondiger.cu*** (Program that determines deposition energies and probability in nanostructured material)
  ○ ***Schrondiger_modif.cu*** (Program that determines deposition energies and probability using equation modified)
- CUDA Toolkit Develop, Optimize, and Deploy GPU-Accelerated Apps. Files ending in *. pbd can be run via the CUDA(R) toolkit, without the following files:
  ○ ***Cristal_00.pdb*** (AISI-1020 Steel Molecule Bond Lengths)
  ○ ***Cristal_01.pdb*** (AISI-1020 Steel Molecule Bond Lengths with NNP's)
  ○ ***Cristal_02.pdb*** (AISI-1020 Steel Molecule Bond Lengths with NNP´s optimize)
  ○ ***Oxido_cromo.pdb*** (Chrome oxide Molecule Bond Lengths)

### 2.2 Modify and View the COMSOL® Program Files

Having a license and an installed program is mandatory. The COMSOL® platform is a simulation platform for electrochemical processes and fluid mechanics.

The files are located at https://github.com/Paco1901/Schrodinger_electrochemistry.git, identified with the *.mph extension:

  ○ ***Electrodeposition.mph*** (Dynamic computer simulation of electrodeposition using COMSOL® program)

### 2.3 We are Obtaining Simulation Data

The corresponding codes (*.cu) must be executed to obtain the electrodeposition data. The numerical values can be viewed in any numerical and text data management program available to the researchers, obtaining the files with *.mat extension, which can also be viewed with the *.tx format [6].

  ○ ***Datos_01.mat*** (Numerical data corresponding to the bond lengths between lattice points using Schröringer's equation)
  ○ ***Datos_02.mat*** (Numerical data corresponding to the bond lengths of chromium, iron, manganese and oxygen between lattice points using Schrödinger's equation)
  ○ ***Datos_03.mat*** (Numerical data prediction of diffusion energy between lattice points using modified-Schrödinger equation)
  ○ ***Datos_04.mat*** (Numerical data prediction of electrodeposition energy between lattice points using modified-Schrödinger equation)
  ○ ***Datos_05.mat*** (Numerical data prediction of probability energy electro diffusion between lattice points using modified-Schrödinger equation)
  ○ ***Datos_06.mat*** (Numerical data corresponding of energy between chromium, iron, manganese, and oxygen using Schrödinger-Lenard-Jones)
  ○ ***Datos_07.mat*** (Numerical data corresponding of energy between chromium, iron, manganese, and oxygen using Schrödinger-Lenard-Jones-Nernst-Plank)
  ○ ***Datos_08.mat*** (Numerical data corresponding of deposition energy chromium, iron, manganese, and oxygen using Schrödinger-Lenard-Jones)
  ○ ***Datos_09.mat*** (Numerical data corresponding of quantum energy between chromium, iron, manganese, and oxygen using Schrödinger-Nernst-Plank)
  ○ ***Datos_10.mat*** (Numerical data probability of electrodeposition-electro-diffusion energy, using equation modified-Schrödinger-Lernard-Jones-Nernst-Plank)

### 2.4 Base Data for Simulation of Coating-Substrate Arrangements

The source codes request the data to be entered before the simulation process at the beginning of the execution; the data request is textual and corresponds to the parameters shown in Table 1.

**Table 1:** Description of the coating-substrate arrangements.

Arrangement	Coating (Supply material)	Substratum
CrNPs -AISI 1020	CrNPs in Cr ^0^ and Cr ^+3^ states, average size 40 nm, preferred spherical shape.	Material and composition: See table 3 in the original reference article [2]. Size: 1cm2 ^(^1cmX1cm)
CrNPs-A68		

The code requests the data only as magnitude; during execution, the corresponding units are considered within the code as base units, for example, voltage (V), thickness (nm), etc.

The interpretation of the data and the results are published in the original article published in the Chemical Engineering Science Magazine [2].

## 3 Experimental Design, Materials and Methods

Practical and detailed information on the use of the data and the configuration for the simulations is shown below. The formation and growth of the chromium coating formed on a steel surface were analyzed using the model shown in Eq. 1 implemented in CUDA® and COMSOL® software and using a supercell model for atomic interaction simulation.

### 3.1 Preparation of Coating-Substrate Arrangements

The electrodeposition mechanism was analyzed using two coating-substrate arrangements of chromium nanoparticles (CrNPs) and commercial steel substrates, see Table 1.

A computational theoretical model was applied for the analysis that uses a modified Schrödinger equation combined with the Lenard -Jones equation and the Nernst-Planck equation. From the quantum mechanics (QM) perspective, the resulting model allows us to predict the electrodeposition capacity in material systems considering electronic stability, interdiffusion, and the minimum potential required for the process. The model used is shown in Eq. (1).

(1) $$\begin{array}{ccc} i\hbar \frac{\partial }{\partial t}\left(-D\left(\nabla \cdot C+\frac{zF}{RT}C\nabla \cdot \phi \right)\right) & = & -K_{\propto }{\left(\hbar \nabla \right)}^{\propto }\left(-D\left(\nabla \cdot C+\frac{zF}{RT}C\nabla \cdot \phi \right)\right)\\ & & +\;4\varepsilon \left[{\left(\frac{\sigma }{x}\right)}^{12}-{\left(\frac{\sigma }{x}\right)}^{6}\right]\left(-D\left(\nabla \cdot C+\frac{zF}{RT}C\nabla \cdot \phi \right)\right) \end{array}$$

The description of the related variables in the model, as well as their corresponding units, can be consulted in the reference article [2].

The discretization of the model for the simulation process, using the finite element method (FEM) and considering the electric potential and electroneutrality equations with and without the inclusion of quantum leap, is shown in Eq. (2).

(2) $$\begin{array}{ccc} & & i\hbar \left\{\sum_{j=1}^{m}\left[\frac{1}{\Delta t}\int_{\Omega }\varphi_{j}\varphi_{i}d\Omega -\frac{z_{\propto }D_{\propto }F}{RT}\sum_{k=1}^{m}\phi_{k}^{n}\left(\int_{\Omega }\varphi_{j}\nabla \varphi_{k}\cdot \nabla \varphi_{i}d\Omega \right)\right]C_{\propto j}^{n}\right\}\\ & & \;=-K_{\propto }{\left(\hbar \nabla \right)}^{\propto }\left\{\sum_{j\;=\;1}^{m}\left[\frac{1}{\Delta t}\int_{\Omega }\varphi_{j}\varphi_{i}d\Omega -\frac{z_{\propto }D_{\propto }F}{RT}\sum_{k\;=\;1}^{m}\phi_{k}^{n}\left(\int_{\Omega }\varphi_{j}\nabla \varphi_{k}\nabla \varphi_{i}d\Omega \right)\right]C_{\propto j}^{n}\right\}\\ & & \;+\;4\varepsilon \left[{\left(\frac{\sigma }{x}\right)}^{12}-{\left(\frac{\sigma }{x}\right)}^{6}\right]\left\{\sum_{j=1}^{m}\left[\frac{1}{\Delta t}\int_{\Omega }\varphi_{j}\varphi_{i}d\Omega -\frac{z_{\propto }D_{\propto }F}{RT}\sum_{k\;=\;1}^{m}\phi_{k}^{n}\left(\int_{\Omega }\varphi_{j}\nabla \varphi_{k}\nabla \varphi_{i}d\Omega \right)\right]C_{\propto j}^{n}\right\} \end{array}$$

### 3.2 Parameters for QM Simulations and Commands for Code Execution

**Fig. 2:**
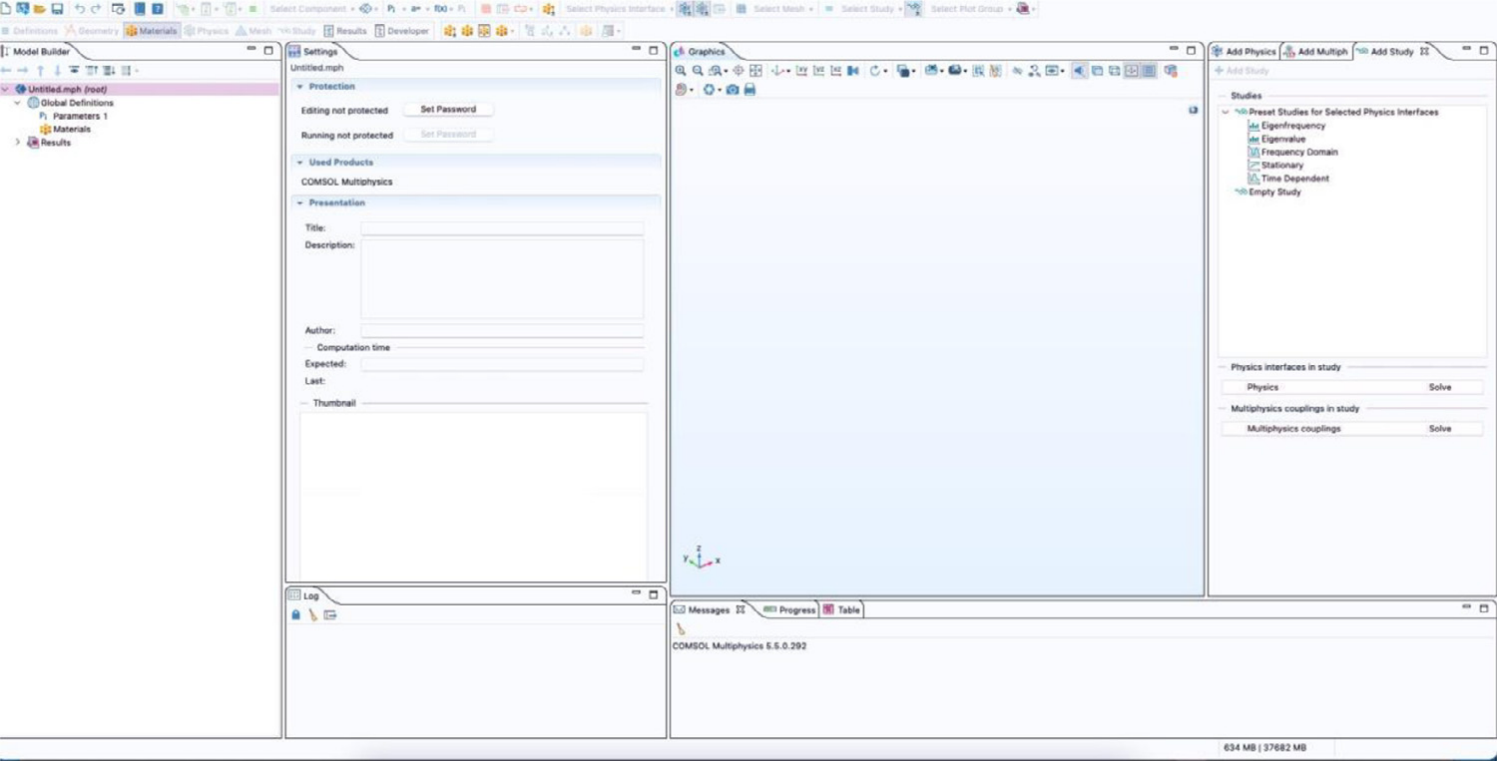
COMSOL® environment.

The following parameters are introduced in the codes in *.cu and *.m, for the endings *.mph is developed in a windowed environment (Fig. 2).

C:\\>star_chrome/unknow/www.github.com/Paco1901/Schrodinger_electrochemistry.git/Schrodinger.cu

User_LNS_ 1:Schrondiger.cu/*.*.exe;

>>V=0.5

>>x=0.0001

>>y=0

>>z=0

>>Type: Cr

>> Substrate: FeMnO

User_LNS_ 2:Schrondiger.exe/save//Data01.mat

User_LNS_3:save 'Data01.mat'/host_UPAEP_Paco1901/*.*.kernel

User_LNS_ 4:dowload'Data 01.mat'/host_UPAEP_Paco1901/save(c:\\*.*.mat

For the environment in COMSOL®

C: \>star_chrome/LNS_fcosanchez/host_234548898/comsol/electrodos.mph/1//2//34

### 3.3 Commands for the Execution of the Codes

The following commands are implemented to determine the global solution of the Schrödinger equation for the determination of deposition and electrodeposition [[7], [8]].

*Schrödinger equation*
C:\>start_chrome/unknow/www.github.com/Paco1901/Schrodinger_electrochemistry.git /Schrodinger.cu//kernel_global_solution
*Electrodepositing Potential*
C:\>start_chrome/unknow/www.github.com/Paco1901/Schrodinger_electrochemistry.git /Schrodinger_modif.cu//kernel_global_solution_modif
*Energy Deposition*
C:\>start_chrome/unknow/www.github.com/Paco1901/Schrodinger_electrochemistry.git /Schrodinger_deposition.cu//kernel_global_solution_deposition
*Nesrt–Plank equation*
C:\>star_chrome/LNS_fcosanchez/www.github.com/Paco1901/Schrodinger_electrochemistry.git /Schodringer_Nest.cu/kernel_LNS_host_0023

### 3.4 Visualization of Coating Formation and Growth

of the structures and energy of each one of the simulations are executed through the following command lines; the visualization language is MATLAB® 2021.

The different figures can be displayed by executing the following command for the Windows 10 operating system.

Electrowinning energy shows the distribution of energy in the (x,y) direction as well as in the vector (x,y,z) directions.

C:\>star_chrome/ www.github.com/Paco1901/Schrodinger_electrochemistry.git /matlab/load ('github/Paco1901/Schrodinger_electrochemistry/Data_01′)/waterfall('Data_01(:,1,2))/plot(' *Energy electrodeposition','Data_01′)*

C:\>star_chrome/ www.github.com/Paco1901/Schrodinger_electrochemistry.git /matlab/load ('github/Paco1901/Schrodinger_electrochemistry/Data_02′)/probplot('Data_02(:,3,4)) /plot(' *Energy electrodeposition','Data_02′)*

C:\>star_chrome/ www.github.com/Paco1901/Schrodinger_electrochemistry.git /matlab/load ('github/Paco1901/Schrodinger_electrochemistry/Data_03′)/lineplot('Data_03(:,3,2)) /plot(' *Energy electrodeposition','Data_03′)*

*The visualization of the change in the potential charge is determined by means of the commands of the following lines.*

C:\>star_chrome/ www.github.com/Paco1901/Schrodinger_electrochemistry.git /matlab/load ('github/Paco1901/Schrodinger_electrochemistry/Data_04′)/probplot('Data_04(1,:,3)) /plot(' *Modified charge potential','Data_04′)*

The commands represent the results obtained as a function of time,

C:\>star_chrome/ www.github.com/Paco1901/Schrodinger_electrochemistry.git /matlab/load ('github/Paco1901/Schrodinger_electrochemistry/Data_05 ')/ probplotk('Data_05(:,:,2)) / plot (' *5 min','Data_05′)*

C:\>star_chrome/ www.github.com/Paco1901/Schrodinger_electrochemistry.git /matlab/load ('github/Paco1901/Schrodinger_electrochemistry/Data_06′)/waterfall('Data_06(:,2,3)) /plot(' *10 min','Data_06′)*

C:\>star_chrome/ www.github.com/Paco1901/Schrodinger_electrochemistry.git /Matlab/load ('GitHub/Paco1901/Schrodinger_electrochemistry/Data_07′)/probplot('Data_07(:,3,4)) /plot(' *5 min','Data_07′)*

For the visualization of the electrodeposition energy, it is obtained in a three-dimensional plane (a); for the deposition energy, it is obtained using the command (b) in the same way in a three-dimensional space, for the electrodiffusion in the line in the same three-dimensional space (c).

(a) C:\>star_chrome/ www.github.com/Paco1901/Schrodinger_electrochemistry.git /matlab/load ('github/Paco1901/Schrodinger_electrochemistry/Data_08′)/waterfall('Data_08(:,1,2)) /plot (' *Electrodeposition energy','Data_08′)*
(b) C:\>star_chrome/ www.github.com/Paco1901/Schrodinger_electrochemistry.git /matlab/load ('github/Paco1901/Schrodinger_electrochemistry/Data_09′)/waterfall('Data_09(:,1,2)) /plot (' *Deposition energy','Data_09′)*
(c) C:\>star_chrome/ www.github.com/Paco1901/Schrodinger_electrochemistry.git /Matlab/load ('GitHub/Paco1901/Schrodinger_electrochemistry/Data_10′)/waterfall('Data_10(:,1,2)) /plot (' *Electro-diffusion','Data_10′)*

## Ethics Statements

The authors declare that the work does not involve human subjects, animal experiments, or data collected from social media platforms, being exempt from an ethical approval process.

## CRediT authorship contribution statement

**G. Rosano-Ortega:** Conceptualization, Methodology, Visualization, Formal analysis, Supervision, Writing – original draft. **M. Bedolla-Hernández:** Conceptualization, Methodology, Investigation, Writing – original draft. **F.J. Sánchez-Ruiz:** Conceptualization, Methodology, Investigation, Visualization, Software, Data curation. **J. Bedolla-Hernández:** Investigation, Writing – review & editing. **P.S. Schabes-Retchkiman:** Investigation, Writing – review & editing. **C.A. Vega-Lebrún:** Project administration, Funding acquisition. **E. Vargas-Viveros:** Investigation, Resources, Writing – review & editing.

## Declaration of Competing Interest

The authors declare that they have no known competing financial interests or personal relationships that could have appeared to influence the work reported in this paper.

## Data Availability

Paco1901/Schrodinger_electrochemestry (Original data) (GitHub).

## Appendix Supplementary Materials

Image, application 1
