# Supplementary material for: Source code and simulation data for the prediction of the electrodeposition mechanism of nanostructured metallic coatings
Source: Data Brief. 2023 May 26;48:109269. doi: 10.1016/j.dib.2023.109269 (PMC10294093; doi:10.1016/j.dib.2023.109269)
Supplement: Supplementary file 1 [file mmc1.pdf]

Make your data count with Data in Brief

Elsevier Journals <STMJournals@author.email.elsevier.com>
Mié 28/09/2022 9:43
Para: Marcos Bedolla Hernández <marcos.bh@apizaco.tecnm.mx>

Elsevier

If you are unable to view this message correctly, click here

Call for Papers

Data in Brief

cover

Congratulations on your recently published article in *Chemical Engineering Science*. Do you know that you can get an additional peer-reviewed publication while also increasing the visibility of your research by publishing a data article in *Data in Brief?*

Data in Brief is a multidisciplinary, open access, peer-reviewed journal, which publishes short, digestible data articles that describe and provide access to research data. By making data and the associated research more discoverable, the journal contributes to open science and improves the reproducibility of your research. It also opens doors for future collaboration and reduces duplication of effort. The journal welcomes submissions from all subject areas.

Make your data count. Submit your paper today.

Twitter

Data in Brief is an open access journal. At Elsevier, finding the right open access home for your research is easy. Find out more.

Research Elements

How do you ensure you are submitting to a trusted journal? Visit Think-Check-Submit

Elsevier

Author Services - Services & Solutions is a communication type sent to you by Elsevier STM Journals.
Unsubscribe from this communication type.

Change your marketing email preferences on the Elsevier Preference Center

Copyright © 2022 Elsevier Limited All rights reserved. | Elsevier Privacy Policy
Elsevier Limited, The Boulevard, Langford Lane, Kidlington, Oxford OX5 1GB UK
